# Mapping *Anopheles stephensi* midgut proteome using high-resolution mass spectrometry

**DOI:** 10.1016/j.dib.2018.02.028

**Published:** 2018-02-21

**Authors:** Ajeet Kumar Mohanty, Gourav Dey, Manish Kumar, Sreelakshmi K. Sreenivasamurthy, Sandeep Garg, T. S. Keshava Prasad, Ashwani Kumar

**Affiliations:** a ICMR-National Institute of Malaria Research, Field Unit, Campal, Panaji, Goa 403001, India; b Institute of Bioinformatics, International Tech Park, Bangalore 560066, India; c Center for Systems Biology and Molecular Medicine, Yenepoya Research Center, Yenepoya (Deemed to be University), Mangalore 575018, India; d Manipal Academy of Higher Education, Madhav Nagar, Manipal, 576104, India; e Department of Microbiology, Goa University, Taleigao Plateau, Goa 403206, India

## Abstract

*Anopheles stephensi* Liston is one of the major vectors of malaria in urban areas of India. Midgut plays a central role in the vector life cycle and transmission of malaria. Because gene expression of *An. stephensi* midgut has not been investigated at protein level, an unbiased mass spectrometry-based proteomic analysis of midgut tissue was carried out. Midgut tissue proteins from female *An. stephensi* mosquitoes were extracted using 0.5% SDS and digested with trypsin using two complementary approaches, in-gel and in-solution digestion. Fractions were analysed on high-resolution mass spectrometer, which resulted in acquisition of 494,960 MS/MS spectra. The MS/MS spectra were searched against protein database comprising of known and predicted proteins reported in *An. stephensi* using Sequest and Mascot software. In all, 47,438 peptides were identified corresponding to 5,709 *An. stephensi* proteins. The identified proteins were functionally categorized based on their cellular localization, biological processes and molecular functions using Gene Ontology (GO) annotation. Several proteins identified in this data are known to mediate the interaction of the *Plasmodium* with vector midgut and also regulate parasite maturation inside the vector host. This study provides information about the protein composition in midgut tissue of female *An. stephensi,* which would be useful in understanding vector parasite interaction at molecular level and besides being useful in devising malaria transmission blocking strategies. The data of this study is related to the research article “Integrating transcriptomics and proteomics data for accurate assembly and annotation of genomes”.

**Specifications Table**

**Table:** 

Subject area	Biology
More specific subject area	Mosquito proteomics
Type of data	Table, Graph, Figure
How data was acquired	LTQ-Orbitrap Velos and LTQ-Orbitrap Elite mass spectrometer (Thermo Scientific)
	Proteome Discoverer 2.1and MASCOT search engine (Matrix Science, London, UK; version 2.2)
	Protein database *An. stephensi* Indian strain (www.VectorBase.org, release date 25th February 2014)
Data format	Analyzed output data
Experimental factors	Midgut tissues were obtained from the laboratory reared female *An. stephensi* mosquitoes
Experimental features	In-gel and in-solution trypsin digestion of proteins followed by LC–MS/MS analysis using LTQ-OrbitrapVelos and LTQ-Orbitrap Elite mass spectrometer (Thermo Scientific).
Data source location	Goa and Bengaluru, India
Data accessibility	Data are available here and via a web application ProteomeXchange Consortium (http://proteomecentral.proteomexchange.org) via the PRIDE partner repository with the dataset identifier PXD001128.

**Value of the data**

• This data set is the largest catalogue of proteins identified from the midgut tissue of female *An. stephensi.*
• Data provides information about midgut proteins involved in different biological and molecular functions, immunity and vector parasite interaction. Overall it enables better understanding of mosquito-parasite interaction and malaria transmission.
• This data could be utilized in future for the development of novel targets for control of disease transmission.

## 1 Data

Presented here is the processed data corresponding to the proteomic analysis of midgut tissue of female *Anopheles stephensi* [1]. The processed data set contains 494,960 MS/MS spectra, which led to identification of 47,438 peptides and 5,709 proteins. All the proteins and peptides identified in this study are listed in Supplementary Table S1 and S2. A total of 127 proteins, which play important roles in vector immunity, have been identified in midgut of female *An. stephensi* mosquitoes. Another 39 proteins, known to be involved in parasite development in the vector, were also identified in this study [2]. Of these, 16 proteins were found to be agonistic in nature thus support *Plasmodium* development in the mosquito host (Table 1) and 23 proteins were found to be antagonistic in nature hence inhibit *Plasmodium* development in mosquito host (Table 2). The Gene Ontology annotation for all the identified proteins were fetched from the VectorBase database [3]. Protein-protein interaction networks were mapped using STRING (version 1.1.0).

**Table 1:** List of agonistic proteins identified which support *Plasmodium* development in mosquito midgut.

**S. No**	***An. stephensi*****ID (Indian strain)**	**Genename**	**Corresponding*****An. gambiae*****ortholog ID**	**Protein description**
1	*ASTEI04504*	*CPR*	*AGAP000500*	Cytochrome P450 reductase
2	*ASTEI00999*	*GSTT1*	*AGAP000761*	Glutathione-S-transferase theta-1
3	*ASTEI00038*	*OXR1*	*AGAP001751*	Oxidation Resistance gene 1
4	*ASTEI00150*	*RFABG*	*AGAP001826*	Retinoid and fatty-acid binding glycoprotein, also known as lipophorin or ApoII/I
5	*ASTEI07966*	*SDR1*	*AGAP002521*	Short-chain dehydrogenases/reductases
6	*ASTEI08473*	*PGRPLC*	*AGAP005203*	PGN Recognition Protein LC
7	*ASTEI02525*	*OXT1*	*AGAP005811*	Peptide-O-xylosyltransferase 1
8	*ASTEI06370*	*PRS1*	*AGAP006102*	*Plasmodium* responsive salivary 1
9	*ASTEI10301*	*Caspar*	*AGAP006473*	Caspar
10	*ASTEI08642*	*ANT*	*AGAP006782*	Adenine nucleotide translocator
11	*ASTEI08607*	*SRPN2*	*AGAP006911*	Serine protease inhibitor 2 (also known as serpin 2)
12	*ASTEI01530*	*LANB2*	*AGAP007629*	Laminin gamma 1
13	*ASTEI07737*	*CP*	*AGAP007864*	F-actin capping protein
14	*ASTEI07671*	*Cactus*	*AGAP007938*	Cactus
15	*ASTEI03999*	*DUOX*	*AGAP009978*	Dual oxidase
16	*ASTEI08424*	*IMPer*	*AGAP013327*	Immunomodulatory peroxidase

**Table 2:** List of antagonistic proteins identified which inhibit *Plasmodium* development in mosquito midgut.

**S. no**	***An. stephensi*****ID (Indian strain)**	**Genename**	**Corresponding*****An. gambiae*****ortholog ID**	**Protein description**
1	*ASTEI06240*	*STAT*	*AGAP000099*	Signal Transducers and Activators of Transcription
2	*ASTEI00224*	*ApoLp-III*	*AGAP013365*	Apolipophorin-III
3	*ASTEI01099*	*Ciboulot*	*AGAP000235*	Beta thymosin family
4	*ASTEI01142*	*WASP*	*AGAP001081*	Wiskott-Aldrich syndrome protein
5	*ASTEI01898*	*ARC P41*	*AGAP008908*	Actin related 2/3 complex 41 KDa subunit P41
6	*ASTEI02221*	*LRIM2*	*AGAP005693*	Leucine-Rich Immune Molecule 2 also known as APL2 or LRRD7
7	*ASTEI02725*	*FADD*	*AGAP007173*	Fas-Associated Death Domain
8	*ASTEI02883*	*LRIM1*	*AGAP006348*	Leucine-Rich Immune Molecule 1
9	*ASTEI03111*	*ARC P21*	*AGAP001712*	Actin related 2/3 complex 21 KDa subunit P21
10	*ASTEI03480*	*GSTT2*	*AGAP000888*	Glutathione-S-transferase theta-2
11	*ASTEI03826*	*REL1*	*AGAP009515*	Relish 1
12	*ASTEI04537*	*IKK-gamma*	*AGAP005933*	Inhibitor of kappa B kinase gamma
13	*ASTEI05239*	*SRPN6*	*AGAP009212*	Serine protease inhibitor 6 (also known as serpin 6)
14	*ASTEI056831*	*CLIPB17*	*AGAP001648*	CLIP-domain serine protease subfamily B17
15	*ASTEI05785*	*MC1*	*AGAP001297*	Mitochondrial carrier 1
16	*ASTEI06809*	*FBN9*	*AGAP011197*	Fibrinogen domain immunolectin 9
17	*ASTEI07221*	*REL2*	*AGAP006747*	Relish - 2
18	*ASTEI07389*	*JNK*	*AGAP009461*	Jun N-terminal Kinase
19	*ASTEI08335*	*GPRFZ2*	*AGAP010442*	Frizzled-2
20	*ASTEI08432*	*TEP1*	*AGAP010815*	Thioester-containing protein 1
21	*ASTEI08922*	*CLIPB4*	*AGAP003250*	CLIP-domain serine protease subfamily B4
22	*ASTEI09290*	*IRSP1*	*AGAP006421*	Infection responsive secreted peptide 1
23	*ASTEI09780*	*LL3*	*AGAP009053*	LITAF-like 3

## 2 Experimental design

### 2.1 Sample preparation

*Female An. stephensi* mosquitoes were obtained from the insectary of ICMR-National Institute of Malaria Research, Field Unit, Goa, where cyclic colony of this mosquito species is maintained at a temperature of 27 ± 2 °C, relative humidity of 70 ± 5% and a photoperiod: scotoperiod of 12:12 h (light:dark). Midguts were dissected from the 500 female *An. stephensi.* The midguts collected were homogenized in 200 µl of 0.5% SDS using ultrasonication. The extracted proteins were then quantified by Bicinchoninic acid assay (Pierce^®^.Cat#: 23225). The proteins extracted were then subjected to in-gel and in-solution trypsin digestion followed by fractionation on off-gel fractionator and reverse-phase liquid chromatography [1].

### 2.2 In-gel digestion

Two hundred micrograms 200 µgof proteins was resolved on 10% SDS-PAGE gel. The gel was stained using Colloidal Coomassie 33 stain (Invitrogen, Carlsbad, CA). Excess stain was removed by giving multiple washings with 10% methanol. The protein lanes were cut into 22 gel pieces and subjected to in-gel trypsin digestion as described previously [4].

### 2.3 In-solution digestion

Four hundred micrograms of protein was subjected to in-solution trypsin digestion. The trypsin-digested peptide mixtures obtained were divided into two equal parts for further separation by using off-gel fractionator and basic Reverse-Phase Liquid Chromatography (bRPLC). Off-gel fractionator (Agilent 3100) was used for fractionating the trypsin digested peptides. Peptides were separated using IPG strip (pH 3–10) by focusing for 50 kVh with maximum current of 50 µA and maximum voltage set to 4000 V. After fractionation, a total of 12 fractions were collected and acidified using 1% TFA and stored at − 80 °C until LC-MS/MS analysis. The remaining digested peptides were fractionated by using bRPLC approach. Peptides were resolved using solvent B (10 m M triethyl ammonium bicarbonate, pH 8.5 in 95% Acetonitrile) with a gradient of 5–60% and 1 ml flow rate per minute for over 60 min. Ninety six fractions were collected using automatic fraction collector, which were further concatenated to 24 fractions, vacuum dried and stored in − 80 °C freezer until further LC-MS/MS analysis as previously described [1].

### 2.4 Mass spectrometry analysis

**Fig. 1:**
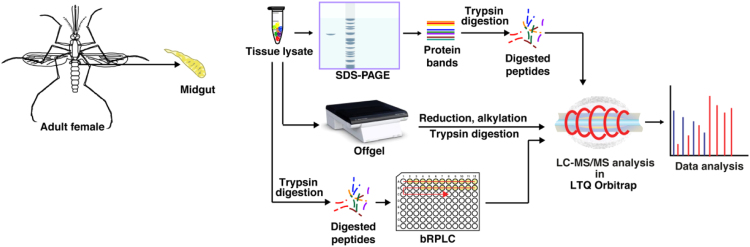
The workflow illustrating the steps involved in proteomic analysis of midgut of female *An. stephensi.* Proteins were extracted from the midgut tissues and then subjected to SDS-PAGE, OFFGEL and bRPLC fractionation. Fractions collected were analyzed on analyzed on LTQ-OrbitrapVelos and LTQ-Orbitrap Elite mass spectrometer. Mascot and SEQUEST algorithms were used to perform database searches.

In this study, a total of 58 LC–MS/MS runs, of which, 24 bRPLC fractions were performed on LTQ-Orbitrap Elite (Thermo Scientific, USA) mass spectrometer interfaced with Easy- nano LC II nano flow liquid chromatography system (Thermo Scientific), while the remaining 34 fractions (including in-gel and off-gel fractions) were analyzed on LTQ-OrbitrapVelos mass spectrometer interfaced with Proxeon Easy nLC system (Thermo Scientific, Bremen, Germany). The peptides from each fraction were reconstituted in 0.1% formic-acid and loaded on pre-column (75 µ × 2 cm) packed with magic C18 AQ (MichromBio-resources, USA) 5 µ particle and 100 Å pore size at flow rate of 5 µl per minute. Peptides were resolved at 250 nl/min flow rate using a linear gradient of 10–35% solvent B (0.1% formic acid in 95% Acetonitrile) over 75 min on an analytical column, of 75 µ × 60 cm, 5 µ particle and 100 Å pore size for Elite and 75 µ × 15 cm, 3 µ particle and 100 Å pore size for Velos was packed using nitrogen pressure cell at 2500 psi. To reduce the back pressure 60 cm analytical column was operated in a heated insulator at 60 °C temperature using butterfly column heater (Phoenix S&T, Inc. PA, USA) and was fitted on flex ion source that was operated at 2.5 kv voltage (Only for Elite). The analysis on mass spectrometry was carried out in a data dependent manner with a full scans in the range of *m*/*z* 350–2000. Full MS scans were measured at a resolution of 120,000 for Elite and 30,000 for Velos at *m*/*z* 400 [1]. Fifteen to twenty most abundant precursor ions were selected from MS scans and fragmented using higher-energy collisional dissociation (HCD). Fragment ions were acquired at a resolution of 30,000 for Elite and 15,000 for Velos. Singly charged ions were excluded and dynamic exclusion was set to 30 s. The steps involved in the proteomic analysis of midgut tissue using mass spectrometry is shown in Fig. 1.

### 2.5 Data analysis

**Fig. 2:**
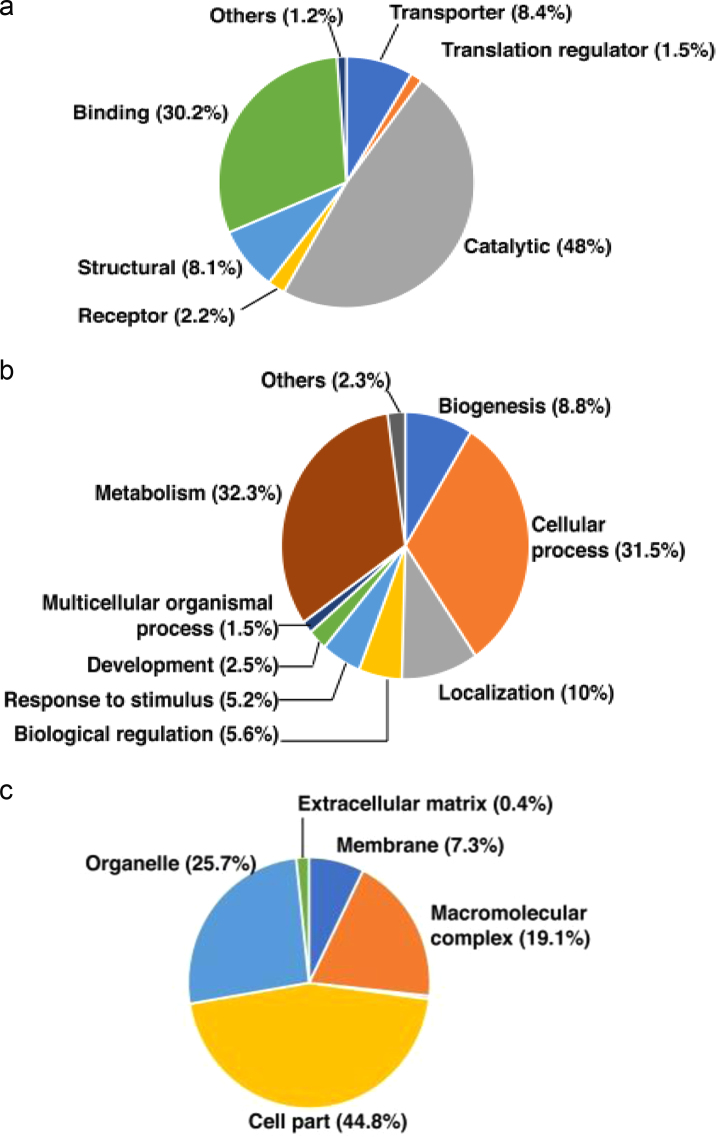
Gene Ontology-based classification of proteins identified from the midgut tissue of female *An. stephensi* mosquito. (A) Molecular functions (B) Biological processes and (C) Cellular localization.

**Fig. 3:**
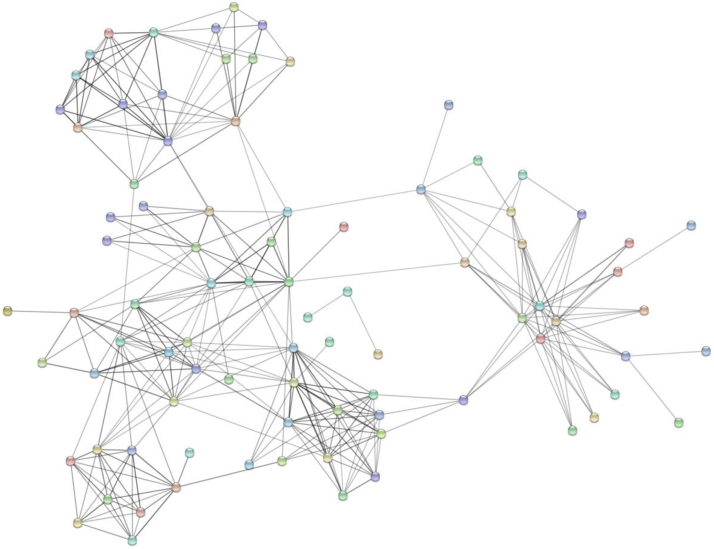
Representation of predicted protein-protein interaction map of proteins identified in female *An. stephensi* midgut. The interaction map was generated using online STRING tool with default parameters. Proteins identified with multiple PSMs and peptides were used of generating the map.

The data obtained was processed using Proteome Discoverer software (version 2.1, Thermo Fisher Scientific, Bremen, Germany) and searched using Sequest and Mascot search algorithm against VectorBase protein database of *An. stephensi,* i.e., Astel2.2. The search parameters included trypsin as the proteolytic enzyme allowing up to two missed cleavages, methionine oxidation was set as a dynamic modification while carbamido-methylation at cysteine was set as static modification. Peptide mass error tolerance and fragment mass error tolerance were set to 20 ppm and 0.1 Da, respectively. The protein and peptide data were extracted with search result parameters as peptide rank one and peptide confidence set as high. For the entire data set, false discovery rate (FDR) was calculated by enabling the peptide sequence analysis using a decoy database and a cut-off of 1% was used for identifications. The identified proteins were functionally categorized based on their subcellular localization, biological processes and molecular function using gene ontology (GO) based annotations available for *An. stephensi* (SDA 500) strain in VectorBase database Supplementary Table S3. Proteins identified were found to be involved in different molecular functions such as catalytic activity (48%), binding activity (30%), transporter activity (8%), structural (8%), receptors (2%) and others (1%). Biological process-based categorization showed that a majority of proteins played a role in metabolism (32%), cellular processes (31%), localization (10%), biogenesis (8%), response to stimulus (5%), biological regulation (5%), development (2%), multicellular organismal process and others (2%). The proteins have been described based on their cellular localization as shown in Fig. 2A–C. The information for *An. stephensi* protein orthologs in *Anopheles gambiae* was fetched using Biomart tool provided through VectorBase Supplementary Table S4. Thirty nine proteins were identified that are known to be involved in parasite development in mosquito. A total of 127 immunogenic proteins were identified using ImmunoDB (http://cegg.unige.ch/Insecta/immunodb/) Supplementary Table S5. The proteins identified were analyzed using online STRING tool to generate an interacting map for all the midgut proteins (Fig. 3, Supplementary Table S6) [5], [6].
